# Supplementary material for: Biodegradation of Petroleum Hydrocarbons by Drechslera spicifera Isolated from Contaminated Soil in Riyadh, Saudi Arabia
Source: Molecules. 2022 Sep 30;27(19):6450. doi: 10.3390/molecules27196450 (PMC9572601; doi:10.3390/molecules27196450)
Supplement: Supplementary file 1 [file molecules-27-06450-s001.zip › molecules-1825529-supplementary.pdf]

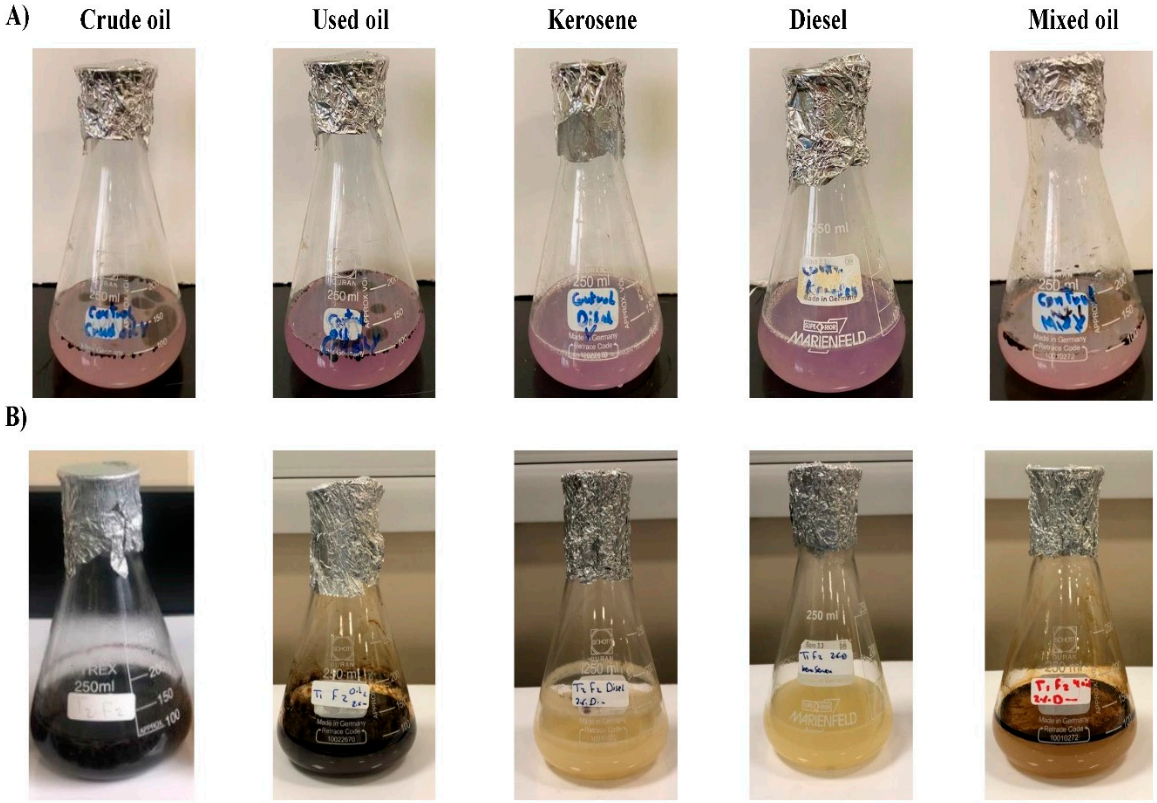


Supplementary Figure S1: DCPIP assay of D. spicifera on liquid media (MSM) containing 1% of different hydrocarbons. Liquid media (MSM) containing 1% of various oil sources; 0.1% (v/v) of Tween 80, and 0.6 mg/mL of redox indicator (DCPIP). A) DCPIP with different oil sources, without *D. spicifera*, as a control, B) DCPIP with different oil sources and *D. spicifera*.

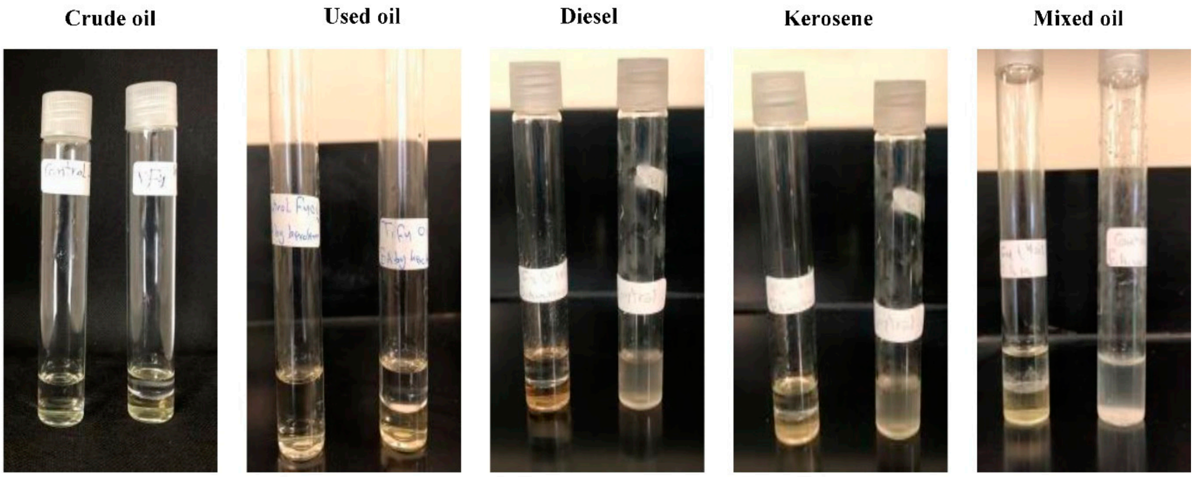


Supplementary Figure S2: Emulsification test of different hydrocarbons. The emulsification activity was measured to test the supernatant (CFS) containing biosurfactants from *D. spicifera.*
